# Fire Proofing Essential in Modern Hospitals

**Published:** 1902-09

**Authors:** 


### FIRE PROOFING ESSENTIAL IN MODERN HOSPITALS.

The following is from the *National Hospital Record:*

"The old saying regarding the locking of the stable door after the horse has been stolen is being exemplified in Chicago as a result of the death of several patients in a sanitarium recently destroyed by fire. A general, and it is supposed rigid, examination is being made of all hospitals in that city for the purpose of ascertaining their provisions for escape in case of fire. According to the newspaper reports, several of the hospitals have been found to possess totally inadequate provisions in this direction, and extensive and radical changes have been ordered. It is to be hoped that the work may be made most thorough, in order that there may be no recurrence of the late disaster. It should be made a criminal offense for any person or persons to operate a hospital or other similar institution that is not fully and practically equipped for the protection of the inmates against fire."

All of which we heartily endorse. It is bad enough to build firetrap hotels, apartment houses and the like, but it is no less than criminal to subject dozens, and perhaps hundreds, of helpless, bedridden hospital inmates to the constant danger of being roasted alive, simply for the sake of saving a few thousand dollars on the cost of building. Last year, in the United States alone, seventy-eight sanatoria and hospitals were destroyed by fire, with a considerable loss of life among the hapless inmates in almost every instance.

With reference to the frequent occurrence of fires in hospitals, asylums, and college dormitories, *The Inland Architect and News Record* says: "Notwithstanding the extreme cheapness of fireproofing material compared with ten years ago, the loss of this class of buildings is increasing year by year." It is remarkable, and yet it is true, that hospitals, as shown by fire records, are more combustible than are even paper mills and factories.

Law and public sentiment should combine to force the builders of such institutions to make adequate provision for protection against fire. R.

